# Role of Echocardiography in Diabetic Cardiomyopathy: From Mechanisms to Clinical Practice

**DOI:** 10.3390/jcdd10020046

**Published:** 2023-01-26

**Authors:** Hrvoje Urlic, Marko Kumric, Josip Vrdoljak, Dinko Martinovic, Goran Dujic, Marino Vilovic, Tina Ticinovic Kurir, Josko Bozic

**Affiliations:** 1 Department of Pathophysiology, University of Split School of Medicine, 21000 Split, Croatia; 2 Clinical Department of Diagnostic and Interventional Radiology, University Hospital of Split, 21000 Split, Croatia; 3 Department of Endocrinology, Diabetes and Metabolic disorders, University Hospital of Split, 21000 Split, Croatia

**Keywords:** diabetes mellitus, diabetic cardiomyopathy, heart failure, echocardiography, diastolic dysfunction, systolic dysfunction, speckle tracking echocardiography, coronary flow reserve

## Abstract

It has been well established that diabetes mellitus (DM) is considered as a core risk factor for the development of cardiovascular diseases. However, what is less appreciated is the fact that DM may affect cardiac function irrespective of cardiac pathologies to which it contributes, such as coronary artery disease and hypertension. Although echocardiography provides accurate and reproducible diagnostic and prognostic data in patients with DM, its use in these patients is still underappreciated, resulting in progression of DM-related heart failure in many patients. Hence, in the present review, we aimed to discuss the role of echocardiography in the contemporary management of diabetic cardiomyopathy (DCM), as well as the role of emerging echocardiographic techniques, which may contribute to earlier diagnosis and more appropriate management of this complication of DM. In order to improve outcomes, focus must be placed on early diagnosis of this condition using a combination of echocardiography and emerging biomarkers, but perhaps the more important thing is to change perspective when it comes to the clinical importance of DCM.

## 1. Introduction

Accounting for more than five million deaths worldwide and with mortality rate of 82.4 per 100,000, diabetes mellitus (DM) has become one of the main global healthcare issues [1]. It has been well established that DM stands as a core risk factor for the development of cardiovascular diseases (CVD) [2]. Moreover, the prevalence of DM in heart failure (HF) could be over 40%, whereas in patients with DM, the prevalence of HF varies between 10% to 22% [3]. To put it in perspective, for each 1% increase in glycosylated hemoglobin (HbA1c), there is an 8% increased risk for HF development. However, what is less appreciated is the fact that DM may affect cardiac function irrespective of cardiac pathologies to which it contributes, such as coronary artery disease (CAD). Rubler et al. coined the term diabetic cardiomyopathy (DCM), back in 1972, after studying autopsy analysis of four diabetics who died of HF, without indication of CAD or any other pathology that could enlighten the spotted structural changes [4,5]. Nowadays, although universal definition does not exist, DCM is usually defined by the presence of abnormal myocardial architecture and malfunctioning, without any evidence of existing cardiovascular risk factors, such as CAD, hypertension, and significant valvular disease among individuals with DM [6]. Diastolic dysfunction (DD) is considered to be an early sign of this diabetic myocardial disease, developing prior to the systolic dysfunction. In consideration of its long asymptomatic phase, and the fact that most patients with long-standing DM have CAD and hypertension, it is still demanding to diagnose DCM in everyday clinical practice. Echocardiography is a valuable, time-saving, and cost-efficient tool that can be performed at a patient’s bedside. It also provides accurate and reproducible diagnostic and prognostic data in patients with DM [7]. However, it must be acknowledged that echocardiography can sometimes yield undependable values due to inter-vendor differences, inter-observer variability, and numerous clinical states that may affect image quality [8]. In the contemporary view of DCM, echocardiographic findings that imply the presence of DCM are the presence of DD, with or without reduced left ventricular ejection fraction (LVEF). Nevertheless, potential of echocardiography in early diagnosis and management of these patients has been underappreciated, thus resulting in poorer outcomes. Hence, in the present review, we aimed to discuss the role of echocardiography in the contemporary management of DCM, as well as the role of emerging echocardiographic techniques, which may contribute to earlier diagnosis and more appropriate management of this complication of DM [9].

## 2. Pathophysiology of Diabetic Cardiomyopathy

DM is a multifactorial metabolic disorder, which leads to issues concerning increased risk of microvascular and macrovascular pathologies. However, hyperglycemia and other pathophysiological mechanisms operating in DM, such as insulin resistance, dysregulation of sympathetic nervous system (SNS), renin-angiotensin-aldosterone system (RAAS), metabolic disequilibrium, increased oxidative stress, impaired calcium handling, microvascular dysfunction, inadequate immune response, abnormal subcellular function, and accumulation of advanced glycation end-products (AGEs), seem to affect cardiac function irrespective of macrovascular CAD [10,11]. For instance, left ventricular hypertrophy, an ominous predictive sign, and an autonomous risk factor for CV incidents, is commonly a consequence of hyperglycemia operating in type 2 DM patients [12].

In addition to serving as capacitance vessels holding 90% of the myocardial blood volume, the coronary microcirculation is the major source of regulation of myocardial blood flow, a role which becomes vital in the presence of a stenosis where coronary autoregulation is required to maintain flow [13]. Those vessels appear to be diminished in DM, facilitated by multiple pathological processes [14]. Rigidity of microcirculation is ordinarily detected within DM population. It is mainly caused by hyperinsulinemia-stimulated vascular smooth muscle cells alteration to an osteoblast-like phenotype [15]. DM type 2 is characterized by insulin resistance and, consequently, higher levels of reactive oxygen species, which contribute to development of endothelial dysfunction by hindering NO synthesis [16,17]. In this regard, an emerging mechanism that may in part explain progression to HF in patients with DM is endothelial to mesenchymal transition (EndoMT), a mechanistic phenomenon, which enlights the deficit of physiological vascular phenotype of endothelial cells and multiplicated cardiac fibroblast matter in diabetic heart [18]. EndoMT is a unique form of epithelial-to-mesenchymal transition regarded as loss of the endothelial characteristics and the acquirement of fibroblast, mesenchymal, or stem-cell-like features [19]. Even though the accurate order of incidents continues to be controversial, endothelial cells undergoing EndoMT drop the expression of typical surface endothelial markers, which results in disruption of organized compact cell layer [20]. This harmful cascade may be a crucial provider to the onset of heart failure with preserved ejection fraction (HFpEF), which is now supported with studies that include treatment of HFpEF with SGLT2 inhibitors [21]. Modified sympathetic nervous system response is a distinctive feature of DM and it is presented with higher expression of β1-adrenergic receptors linked with myocyte apoptosis and hypertrophy [22]. Cardiac autonomic neuropathy (CAN) is one of the established hallmarks of DM, and it results from interactions between disease length, neuronal death, arterial blood pressure, aging, and most importantly, glycemic regulation [23,24]. Hyperglycemia is considered to be an initial culprit in CAN development. It boosts mitochondrial production of oxygen radicals, thus triggering oxidative stress damage to the microvasculature, which distributes these peripheral nerves [24]. Various research indicates that hyperglycemia enhances the transcription of angiotensinogen and consequently angiotensin II, therefore enhancing the RAAS activity [25]. Hence, obesity, resembling an oversupply state, is also linked with intensification of the RAAS impact [26]. Furthermore, RAAS activity stimuli insulin signal transduction pathways on various stages result in plenty of cardiovascular and peripheral consequences [27]. Finally, an essential factor contributing to DD is decreased calcium pump activity, which is induced due to insufficient sequestration of sarcoplasmic reticulum calcium [10].

**Figure 1:**
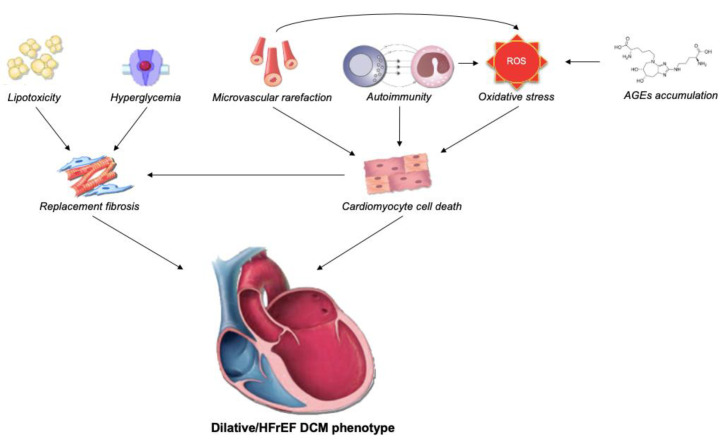
Pathophysiological mechanisms underlying the “dilative” diabetic cardiomyopathy phenotype. Complex interaction of hyperglycemia, lipotoxicity, oxidative stress, autoimmune cell destruction, hypoxia, and AGE deposition leads to cardiomyocyte cell death and replacement fibrosis, with end-result being reduced contractile force and clinical syndrome of HFrEF. Abbreviations: AGEs: advanced glycation end products; DCM: diabetic cardiomyopathy; HFrEF: heart failure with reduced ejection fraction; ROS: reactive oxygen species.

**Figure 2:**
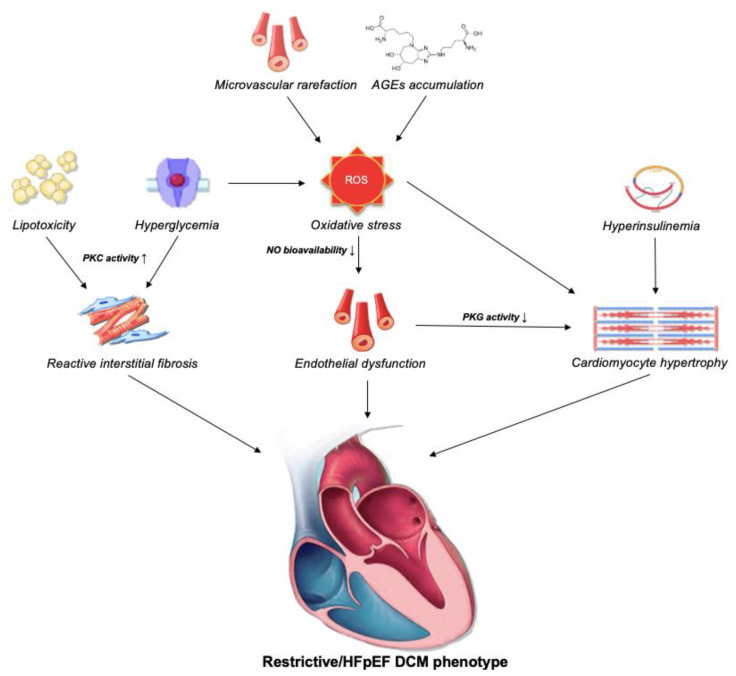
Pathophysiological mechanisms underlying the “restrictive” DCM phenotype. Hyperglycemia, lipotoxicity, AGE accumulation, and microvascular rarefaction all contribute to the development of endothelial dysfunction. Subsequently, lack of NO bioavailability leads to myocardial hypertrophy by down-regulation of PKG activity, which is further supported by hyperinsulinemia. Finally, pathological hypertrophy dampens diastolic function of the myocardium. On the other hand, hyperglycemia and lipotoxicity increase PKC activity, thus leading to reactive interstitial fibrosis that further dampens diastolic function and contributes to the development of HFpEF. Abbreviations: AGEs: advanced glycation endproducts; DCM: diabetic cardiomyopathy; HFpEF: heart failure with preserved ejection fraction; PKG: protein kinase G; PKC: protein kinase C; ROS: reactive oxygen species.

DCM is generally asymptomatic during most of the disease course [10]. However, as symptoms of HF start to emerge, prognosis of patients suddenly deteriorates [28]. During this long asymptomatic period, the aforementioned mechanisms operate (with variable contribution), thus dampening both systolic and diastolic function. Multiple authors aimed to delineate progression of DCM, but with limited success. In a seminal paper by Seferovic and Paulus, the authors advocate that DCM may develop in two distinct phenotypes, depending on the prevailing mechanisms [29]. The first phenotype, consistent with original DCM description, resembles that of dilative cardiomyopathy/HFrEF (eccentric LV remodeling and systolic LV dysfunction). On a microscopical level, dilated phenotype is characterized by loss of sarcomeres and replacement fibrosis, and the principal driving mechanisms seem to be cardiomyocyte cell death as a result of oxidative stress, microvascular rarefaction, inflammation, and AGEs deposition (Figure 1) [30]. The second phenotype resembles that of restrictive cardiomyopathy/HFpEF (preserved EF and severely impaired diastolic function). The restrictive phenotype is characterized by marked hypertrophy and reactive fibrosis, which are products of complex interplay between hyperglycemia, hyperinsulinemia, lipotoxicity, AGEs deposition, and consequent microvascular endothelial dysfunction (Figure 2) [31,32]. Of important note, although EF is preserved in the latter phenotype, as demonstrated by strain analysis, the systolic function in these patients is usually impaired [33].

## 3. The Role of Conventional Echocardiography in DCM

**Figure 3:**
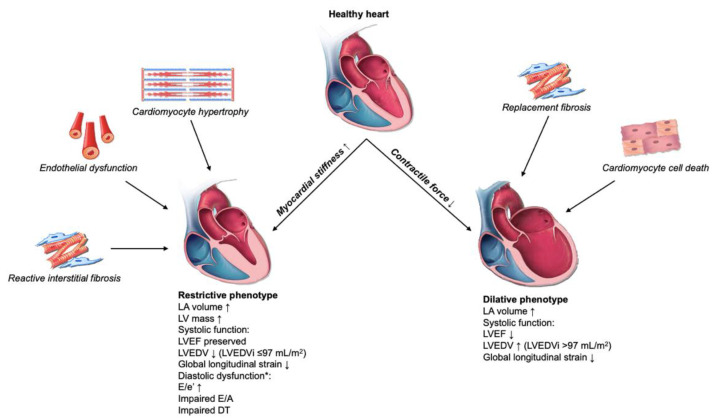
Echocardiographic landmarks of different diabetic cardiomyopathy phenotypes with associated pathophysiologic pathways. Abbreviations: LA: left atrium; LV: left ventricle; LVEF: left ventricular ejection fraction; LVEDV: left ventricular end-diastolic volume; LVEDVi: left ventricular end-diastolic volume index; DT: deceleration time. * E/e’ is increased in most patients, whereas E/A and DT depend on severity of diastolic dysfunction. For instance, Grade I diastolic dysfunction results in reduced E/A ratio, and Grade II and III with normal to high E/A ratio.

Conventional echocardiography is considered to be a primary modality for cardiac imaging that enables precise estimation of cardiac structures and function. Furthermore, it is a non-invasive, painless, and risk-free procedure, which has played a fundamental role in assessment of LV diastolic dysfunction during the last few decades [34,35]. The evaluation of LV diastolic function should be a vital part of a routine echocardiographic examination, especially in patients with symptoms such as progressive fatigue, dyspnea, chest discomfort, etc. Therefore, it is an essential tool for imaging, diagnosing, staging, prognosis, and effective treatment of DCM. Currently, there are several conventional imaging modalities in echocardiography that are beneficial in workup of patients with DCM. Echocardiographic findings in different DCM phenotypes are delineated in Figure 3.

Pulsed wave Doppler (PW Doppler), an imaging modality based on acquirement of the local erythrocyte flow continuum of a precisely defined area in the heart, is one of the main tools for quantification of DD [36,37,38,39]. The first measurements of mitral inflow consist of E wave and A wave velocity, the E/A ratio, deceleration time (DT), diastasis, isovolumic relaxation time (IVRT), and isovolumic contraction time. Secondary measurements consist of mitral A wave duration and the A wave velocity time integral (VTI), diastolic filling time, and total mitral inflow velocity integral, as well as atrial filling fraction [40,41]. The mitral inflow velocities, as well as time intervals, such as DT and IVRT, are altered by changes in LV end systolic and/or end-diastolic volumes, LV elastic recoil, and/or LV diastolic pressures [42]. Mitral E/A and DT are used as a way of establishing mitral inflow patterns. They consist of normal, impaired, or incomplete LV relaxation, pseudo normal LV filling (PNF), as well as restrictive LV filling. However, identifying PNF by only using the mitral inflow velocities may be demanding. Moreover, some fewer common patterns, such as triphasic mitral flow velocity flow patterns, are occasionally noticed. Elderly patients who suffer severe and long-standing arterial hypertension, as well as patients with DCM, hypertrophic cardiomyopathy (HCM), often have the most abnormal diastolic physiology and LV filling pattern variants [43].

Tissue Doppler imaging (TDI) is a valuable method to evaluate global and regional myocardial diastolic, as well as systolic function through cardiac cycle by examining longitudinal component of myocardial contraction [44,45]. The biggest drawback of TDI implementation is its angle dependence. Furthermore, for TDI to be accurate, it requires high frame rates (>100 fps) for image acquisition with extremely good temporal resolution [46]. Another important parameter used to define DD is left atrial (LA) volume. It is calculated during the time of its largest volume, at ventricular end-systole, using the area length method or the modified Simpsons method [47]. In summary, according to recent recommendations for evaluation of left ventricular diastolic function by echocardiography issued by cooperation between leading societies, the fundamental variables proposed for evaluation of LV DD grade include mitral annular e′ velocity (septal e′< 7 cm/s, lateral e′ < 10, E/e′ ratio > 14), mitral flow velocities, LA maximum volume index > 34 mL/m^2^, and peak velocity of tricuspid regurgitant jet > 2.8 m/s. DD is considered present in the case when more than half of the above-noted variables meet the cutoff value. Notably, findings are rendered inconclusive to estimate LA pressure when there is 50% discordance or with only one available variable [48].

The use of two-dimensional and Doppler techniques assists us to systematically assess cardiac function in diabetics, especially those with DCM [49]. Doppler echocardiography had a large contribution in verifying the presence of a distinct diabetic cardiomyopathy. Various studies displayed the evidence of LV remodeling and hypertrophy in change in both LV diastolic filling and systolic function in DM. Furthermore, systolic and diastolic function were not dependent of the coexistence of concomitant risk factors [50]. When it comes to clinically valuable points for obtaining information about LVFP, as well as unmasking Doppler inflow pseudo-normal pattern, the combination of tissue Doppler study of mitral annulus with transmittal inflow is of great help, knowing that pseudo normal pattern may be a hinge point in terms of progressing toward advanced heart failure [51,52]. An early indication of DD is considered to be Doppler patterns of impaired LV relaxation. They are defined by reduced early as well as increased late diastolic flow. Most severe LV decompensation is also associated with more progressive grades, such as those which are manifested by predominant early diastolic filling and rapid velocity deceleration, characteristic in restrictive filling patterns.

When it comes to LVFP rising in order to continue normal cardiac output, as well as increasing the early filling triggered by impaired relaxation, the intermediary and pseudo normal patterns occur. As previously stated, preload dependence is a factor because of which pseudo normal and normal patterns cannot be separated by transmittal inflow. At this stage, in order to more precisely assess DD, an additional analysis of Valsalva maneuver, myocardial velocities, pulmonary venous flow, and LA volume determination is necessary [53].

A somewhat newer, but promising, approach for myocardial mechanical deformation assessment is Speckle-tracking echocardiography (STE). The technique was developed as an alternative to TDI, and permits a more objective and quantitative evaluation of myocardial tissue function [54,55]. It is established by tracking explicit speckle patterns, produced by interferences of ultrasound beams with the myocardial tissue, which then provide advanced diastolic and systolic function assessment. STE can be used to calculate myocardial deformation in three axes (longitudinal, circumferential, and radial strains) [56]. Longitudinal strain embodies the myocardial deformation from the base to the apex, radial strain thickening motions in the radial direction towards the center of the LV cavity, and circumferential strain represents the shortening of LV myocardial fibers along the circular perimeter [57]. A body of data suggests that STE is highly feasible and more reproducible than most of the other available echo-parameters, despite the fact that substantial inter-vendor differences still exist [58]. In addition, the principal advantage of STE in comparison to TDI is its angle independence [59]. Global longitudinal strain (GLS), the most extensively used STE parameter, is now considered robust and highly reliable parameter of systolic function [55]. Specifically, it has been shown that GLS can detect systolic dysfunction much earlier than EF, thus making it interesting for patients with subclinical myocardial damage, such as DCM.

When it comes to systolic function assessment in patients with DCM, the most used and more impaired parameter was GLS, followed by global circumferential strain (GCS) and global radial strain (GRS), respectively. The impairments are possibly due to longitudinally oriented subendocardial fibers, which are the most vulnerable during the early stages of DCM and, therefore, first influenced by the pathological processes [60,61]. The radial and circumferential fibers from subepicardium and mid-myocardium augment and boost their function as response, therefore the only STE variable altered being GLS. During this phase, the subclinical form of DCM is existent, which is evident as HFpEF. During the development of the disease, the mid-myocardium and subepicardium are also altered, which moreover leads to DCM progressing to its clinical phase, as well as HF with reduced ejection fraction [62]. Loncarevic et al. presented more evidence in order to support this direction. Their report showed GLS impairment in all DM patients, regardless whether symptoms, hypertension, or CAD were present, whereas GCS impairment was discovered in cases when DM was related with hypertension or CAD. In general, GLS measurements tend to be more diminished in patients with DM than in healthy controls, and even in patients with prediabetes [63,64]. In fact, studies suggest that GLS is affected by both duration of DM and glycemic control [65]. Moreover, when it comes to DM-patients, GLS is more diminished in those with proteinuria when compared to those without it [66]. Additionally, a similar time-dependent pattern of GLS change is found in patients who suffer from uncomplicated DM type II, which furthermore indicated subclinical systolic dysfunction, also associated with duration and extent of the disease, in patients with diabetes [67]. In a recent sub analysis of the EMPA HEART Cardiolink-6 study, impact of empagliflozin on LV strain (assessed by MRI) has been explored in T2DM patients [68]. Surprisingly, after six-month follow-up, no difference was found between placebo and empagliflozin in any of the strain measurements.

Three-dimensional STE is a recent and less reproducible echocardiographic technique, which also has a high specificity for myocardial dysfunction [69]. Enomoto et al. pointed out the benefits of three-dimensional STE in their study on the correlation between myocardial dysfunction and DM-related microangiopathy [70]. The main benefits of three-dimensional STE in comparison to two-dimensional is a lack of foreshortening effect, and avoidance of potential out-of-plane activity of the echocardiographic speckles [71]. The outcome demonstrated a firm correlation between initial longitudinal systolic dysfunction assessed by GLS and autonomic myocardial neuropathy [70]. Furthermore, Chen et al. pointed out the significance of three-dimensional STE when it comes to evaluating subclinical systolic dysfunction in type 2 DM patients with inadequate glucose blood levels [72]. Finally, a new STE marker—peak systolic longitudinal rotation—was discovered to be diminished in early stages of DCM and, therefore, indicated encouraging perspectives for the upcoming times [73]. 

DM is considered to be one of the fundamental causes of LA remodeling, which also expands the risk of HFpEF, as well as atrial fibrillation. Assessing LA function is permitted by STE measurement even before atrial structural remodeling [74]. Decreased GLS values in LA systolic dysfunction led to the conclusion that that left atrial deformation mechanics are impaired in patients with DM and HFpEF [75]. Furthermore, there is existing evidence of concomitant LA systolic and diastolic dysfunctions in type 2 DM patients with nephropathy and proteinuria [74]. In a study by Cameli et al., peak left atrial longitudinal strain and global atrial-ventricular strain were more suitable at assessing subclinical myocardial dysfunction than GLS in asymptomatic patients with arterial hypertension and DM [76]. Moreover, Mohseni-Badalabadi et al. discussed left atrium longitudinal strain being impaired in obese patients with type 2 DM [77]. Accumulating data suggest that global LA strain is a solid and autonomous predictor of cardiovascular events, offering more prognostic information than conventional parameters, such as indexed LA volume, LA area, or LA diameter, and that level of left ventricular DD is linked to LA strain as well as to the strain rates [78,79]. In line with this, it was also demonstrated for patients with DM that, when compared with controls, longitudinal strain in all six segments of the LA is lower, and LA strain changes overall correspond with other indices of DD in this population [80,81,82].

Various studies pointed out right ventricular (RV) systolic and diastolic dysfunctions in type 1 and type 2 DM patients, as well as RV global and layer-specific strain as an eminent morbidity and mortality predictor [83,84]. Furthermore, LV and RV systolic dysfunction, as well as DD, were related to type 1 DM. In summary, the above-mentioned findings bring evidence for the presence of early systolic dysfunction in DCM, yet perhaps the biggest setback of STE in DCM is low specificity, as various noxae, some of which are not even recognized as such, can result in discrete subclinical impairment of LV function [62,85].

Resting echocardiographic methods are not always successful in enlightening clinical status (most markedly exertional dyspnea and fatigue) in patients which are admitted for reasons other than HF. Firstly, symptoms in question are more likely to be exertional, which means they may reflect an altered physiological status to the one that was noticed during the resting echocardiographic study. Natriuretic peptides have been endorsed by many guidelines, as a tool for such discrimination, yet with limited reliability [85]. Therefore, stress echocardiography is proposed as the test of choice when it comes to determining exertional symptoms in patients with suspected LV dysfunction [86,87]. Whilst exercising, tachycardia reduces the diastolic time length, which leads to less time for diastolic filling of LV. In order to preserve or increase the stroke volume, myocardial relaxation should be more efficient, and LV suction should be exaggerated. Yet, in patients with DM, when heart rate rises during exercise, DD impedes the required increase in myocardial relaxation. Hence, exercise can expose diastolic abnormalities, which are not noticeable during relaxation [88]. In fact, recent studies demonstrated a link between lack of decrease in minimal LV pressure during exercise, also seen as a manifestation of abnormal LV suction, and impaired LV untwisting during exercise [89]. Many patients demanding stress echocardiography present with E/A < 1, enlarged LA, as well as having an undetermined peak tricuspid retrograde pressure drop. In non-compliant LV, a sudden increase in stroke volume cannot be supported without hefty rise in filling pressures. Although the compensatory increase in left ventricular filling pressures (LVFP) in those patients, the respective increase in stroke volume stays inadequate [90]. Hence, the proportion of cardiac output/PCWP at rest, as well as during exercise, may be accepted as a marker for abnormal exercise [91]. In applied terms, PCWP can be determined using the peak TR velocity. In order to evaluate LVFP, E/e’ has been used, however, the link has only been proved moderate in a wide range of patients [92]. Hence, Doppler echocardiographic parameters cannot be of use when it comes to reflecting small dynamic changes in LVFP. On the other hand, a more comprehensive approach combining an increase in trans-mitral E-wave velocity, unchanged e’, increase in TR peak velocity (>3.3 m/s), as well as none or only minor increase in cardiac output during exercise, may be useful for such purpose. Specifically, contemporary guidelines proposed that diastolic stress echocardiography is ultimately seen as impaired when the septal E/e′ ratio is >15, peak tricuspid regurgitant velocity is >2.8 m/s with exercise, and the septal e′ velocity is <8 cm/s at baseline [93]. 

When it comes to the absence of epicardial coronary artery stenosis, the coronary flow reserve (CFR) is the method that may determine the dysregulation of coronary microcirculation, in association with blood sugar levels, insulin resistance, sympathetic dysregulation, endothelial dysfunction, malfunctions of the renin-angiotensin-aldosterone system, as well as LV architecture remodeling or hypertrophy. DD and impairment of CFR possibly have a similar foundation, as well as being associated in DM [94,95]. Although it poses a technical challenge, and may not be feasible in every patient, CFR can be reliably assessed using Doppler echocardiography [96]. Atar et al. found that, in prediabetic and diabetic patients with patent coronary arteries, CFR was substantially reduced in DM bot not in prediabetes [97]. Thus, it seems that this non-invasive method may detect coronary microvascular dysfunction operating in DM, although it should be used judiciously. For instance, LV hypertrophy, a common companion of DM, may in fact diminish CFR as a consequence of diastolic dysfunction, reduced capillary density, and increased resistance to flow [98]. In line with this, detection of ischemia on stress echocardiography in patients with patent epicardial arteries may also reflect microvascular dysfunction, an important, clinically ambiguous entity that portends worse outcomes for patients, poor quality of life, and greater healthcare expenditures [99,100,101]. However, it has to be addressed that standard stress echocardiography has limited sensitivity and specificity in this setting [102,103]. On the other hand, implementation of myocardial contrast echocardiography (MCE), a technique based on rate of replenishment of echocardiographic contrast microbubbles within the ultrasound beam, increases diagnostic accuracy of stress echo in detecting microvascular dysfunction [104].

## 4. Future Perspectives—The Need for Targeting Early Stages

Although there are a large number of imaging tools which can detect metabolic perturbations in myocardium, a lot of them have not yet been employed to study DCM. It is important to note that this task is quite challenging in DCM given the complex pathophysiologic processes that underlie it. The most frequently used imaging tools which calculate such perturbations and their downstream effects are single-photon emission computed tomography (SPECT), positron emission tomography (PET), and magnetic resonance (MR) based approaches (MR imaging, MR spectroscopy and hyperpolarized MR spectroscopy [105,106]. Studies which focused on pharmacological and genetic preclinical models of DM pointed out the consequences DM can have on the myocardial cells. Most of its detrimental effects are caused by hyperglycaemia. Moreover, there are many mechanisms, such as compound interaction of myocardial metabolic remodelling and its downstream harmful outcomes, for example inflammation, cell fibrosis, mitochondrial dysfunction. The above-mentioned processes are not separated, neither by time, nor by coherence, on the contrary, they are dependent on each other and can occur simultaneously, effecting on myocardial structure and function [107]. To effectively implement previously mentioned instrumentations—SPECT, PET, and MR based approaches—these methods will have to compel the use of multimodality imaging that exploits imaging assets of every tool, enhances imaging set of rules, and diminishes hypothetical side effects [108]. These imaging tools may also enable and facilitate the detection and in multiple cases stratification and quantification, of mentioned processes that characterizes metabolic remodelling and cardiac function deterioration in DCM. During the last few years, various studies examined myocardial strain using cardiac magnetic resonance (CMR). The outcome pointed out comparable relations between DCM and myocardial dysfunction as STE, however, with higher costs [109,110].

In light of poor prognosis of DM related HF, and the relative negligence with regards to this issue, echocardiography should serve as a routine clinical tool to establish the presence and severity of DCM. There are numerous advantages of echocardiography techniques, such as feasibility, reproducibility, ease of application (it can be performed at bedside), and cost efficiency. It is nonetheless unfortunate that there is no long-term echocardiography study available when it comes to defining progression of a disease in more detail. Thus, it makes it demanding to distinguish and stage DCM diagnosis, using standard echocardiography. As a result of lacking agreement between experts, the appropriate diagnostic protocol for echocardiographic evaluation of DCM have not yet been determined among clinicians, in spite of real effort [105,111]. The putative diagnostic protocol could be valuable, even essential, for preclinical diagnosis, risk stratification, and therapeutic management of the DCM. Therefore, in order to improve poor results of DM-related HF, new strategies must be incorporated. In an ideal situation, during the early asymptomatic phase and before irreversible myocardial damage occurs, DCM would be identified. Early metabolic myocardial changes, in both human and animal studies, have been successfully recognised using different imaging approaches, such as Phase-MRI, STE, and nuclear imaging. However, apart from STE, which is becoming increasingly available, the use of nuclear imaging and MRI is still largely limited due to price and availability. On the other hand, STE, albeit promising, may not be as feasible when it comes to accuracy in irregular ventricular remodeling and myocardial wall thickening or thinning [106,112]. 

Future research will have to upgrade DCM strategic approach in order to make it more useful for the clinical practice. A mixture of fairly low cost and precise STE and biomarkers which were shown to be useful in this setting, such as soluble suppression of tumorigenesis-2 (sST2) or markers of collagen turnover [107,113]. Furthermore, in order for studies to be valid, and therefore implemented in everyday clinical practice, they have to be well designed and have a reasonably long term follow up. More importantly, the questions have to be ‘’when and whom to test’’ rather than ‘’what to test’’. Considering that around 12% of diabetics suffer from DCM, it is essential to find a predictive scoring classification of recognizing and establishment of patients’ risk of DCM occurrence, since they all are in jeopardy [108,114]. Hence, screening techniques should be included for all patients with recent onset of DM type II. When it comes to DM type I, there is a need for future research in order to reach more valuable conclusions, due to the inconsistency in pathophysiological aspects [109,115]. The principal issue with regards to DCM is the fact that usual practice for patients with DM type II focuses on CAD, inadvertently neglecting that the heart is affected by DM itself. Perhaps this is a result of the restrictive DCM definition, which describes a rarely seen state in which patients with DM are devoid of either hypertension or some form of CAD. Because of this, DCM is usually perceived as a theoretical entity, and not as culprit of many cases of HF. Therefore, the authors of the present review believe that, in future discourse, the crucial step is to address that DM-related cardiac damage occur regardless of whether we named it DCM or not. Regarding the “when to test” question, it is important to diagnose DCM in early stages, before its progression deteriorates heart function. In this regard, it is debated that there is a need for a simple and reproducible algorithm, in which patients with positive screening tests consisted of STE and standard echocardiography, but also biomarkers, will be directed to more expensive but more specific confirmation tests, such as phase-MRI or advanced echocardiographic techniques. This type of approach will be particularly valuable if new more targeted therapies emerge, considering that most of these therapies will be expensive. 

## 5. Conclusions

Although DM is increasingly recognized as a perpetrator of normal cardiac function, even in the absence of coronary artery disease, efforts must be made to transfer this knowledge into clinical practice. Specifically, in consideration of the long asymptomatic phase of DCM, highlight should be put on screening and early diagnosis of this disease. Perhaps the most feasible ways for screening of subclinical DM-related cardiac dysfunction in the future will be echocardiography and serum biomarkers. Among echocardiographic techniques, apart from the well-established indices of diastolic dysfunction obtained using standard echo, advanced echocardiographic techniques, such as STE, CFR, stress echocardiography and three-dimensional echo, may also be helpful in this setting. Nevertheless, future research is needed to establish the most reproducible algorithms and explore the cost–benefit aspect. In this regard, the question remains whether we can affect outcomes of patients with DCM even if we make an early diagnosis. Finally, perhaps the most important message of this review is a need for change in perspective with regards to clinical importance of DCM.

## Data Availability

Not applicable.
